# BOLD signal variability as potential new biomarker of functional neurological disorders

**DOI:** 10.1016/j.nicl.2024.103625

**Published:** 2024-05-31

**Authors:** Ayla Schneider, Samantha Weber, Anna Wyss, Serafeim Loukas, Selma Aybek

**Affiliations:** a Department of Neurology, Psychosomatic Medicine Unit, Inselspital Bern University Hospital, University of Bern, 3012 Bern, Switzerland; b Translational Imaging Center (TIC), Swiss Institute for Translational and Entrepreneurial Medicine, 3010 Bern, Switzerland; c University of Zurich, Psychiatric University Hospital Zurich, Department of Psychiatry, Psychotherapy and Psychosomatics, 8032 Zurich, Switzerland; d Graduate School for Health Sciences (GHS), University of Bern, 3006 Bern, Switzerland; e Institute of Bioengineering, Ecole Polytechnique Fédérale de Lausanne (EPFL), 1015 Lausanne, Switzerland; f Faculty of Science and Medicine, University of Fribourg, 1700 Fribourg, Switzerland

**Keywords:** Conversion disorders, Longitudinal, Prognostic, Biomarker, Insula, Supplementary motor area

## Abstract

**Background:**

Functional neurological disorder (FND) is a common neuropsychiatric condition with established diagnostic criteria and effective treatments but for which the underlying neuropathophysiological mechanisms remain incompletely understood. Recent neuroimaging studies have revealed FND as a multi-network brain disorder, unveiling alterations across limbic, self-agency, attentional/salience, and sensorimotor networks. However, the relationship between identified brain alterations and disease progression or improvement is less explored.

**Methods:**

This study included resting-state functional magnetic resonance imaging (fMRI) data from 79 patients with FND and 74 age and sex-matched healthy controls (HC). First, voxel-wise BOLD signal variability was computed for each participant and the group-wise difference was calculated. Second, we investigated the potential of BOLD signal variability to serve as a prognostic biomarker for clinical outcome in 47 patients who attended a follow-up measurement after eight months.

**Results:**

The results demonstrated higher BOLD signal variability in key networks, including the somatomotor, salience, limbic, and dorsal attention networks, in patients compared to controls. Longitudinal analysis revealed an increase in BOLD signal variability in the supplementary motor area (SMA) in FND patients who had an improved clinical outcome, suggesting SMA variability as a potential state biomarker. Additionally, higher BOLD signal variability in the left insula at baseline predicted a worse clinical outcome.

**Conclusion:**

This study contributes to the understanding of FND pathophysiology, emphasizing the dynamic nature of neural activity and highlighting the potential of BOLD signal variability as a valuable research tool. The insula and SMA emerge as promising regions for further investigation as prognostic and state markers.

## Highlights

• Higher BOLD variability in somatomotor, salience and limbic networks in FND.
• Better clinical outcome at follow-up associated with higher variability in SMA.
• Higher insular variability at baseline predicted a worse clinical outcome.
• BOLD signal variability might present a prognostic and state biomarker for FND.

## 1 Introduction

Functional neurological disorder (FND) is a common medical condition (Bennett et al., 2021, Carson and Lehn, 2016; Hallett et al., n.d.) presenting with diverse neurological symptoms and typically motor or sensory symptom patterns that cannot been explained by an underlying classical neurological disorder (Aybek and Perez, 2022, Espay et al., 2018a). Currently there are well established rule-in diagnostic criteria (Association and Force, 2013; Varley et al., 2023) and multimodal treatment options available (Bennett et al., 2021; Hallett et al., n.d.; LaFaver, 2020, Varley et al., 2023), but our understanding of the neuropathophysiological mechanisms, the underlying development and clinical course of the diverse symptoms remains limited (Drane et al., 2021).

Using diverse neuroimaging techniques, recent research has unveiled FND as a multi-network brain disorder (Drane et al., 2021; Perez et al., 2021). This condition has been characterized by alterations in task-based as well as in resting-state analysis across limbic/salience (Demartini et al., 2021, Hassa et al., 2021, Hassa et al., 2017, Stone et al., 2007, Voon et al., 2011, Weber et al., 2024), self-agency (Baek et al., 2017, Maurer et al., 2016, Voon et al., 2010), attentional (Marapin et al., 2020, Stager et al., 2022) and sensorimotor networks (Aybek et al., 2015; Voon et al., 2011, Weber et al., 2022b). Disturbed self-agency (Baek et al., 2017, Marapin et al., 2020) in FND patients seems to be manifested in a hypoactivation of the right temporal parietal junction (TPJ) and decreased connectivity of the TPJ with limbic and sensorimotor regions in task-based (Voon et al., 2010) as well as resting-state (Maurer et al., 2016) studies that could potentially be reversed by neuromodulation (Bühler et al., 2024). These findings go align with a lower sense of control in a game manipulated agency in children with functional seizure where also a poorer selective attention and cognitive inhibition was reported (Stager et al., 2022). In the limbic network, hyperactivation of the amygdala while exposed to fearful emotional stimuli (Voon et al., 2010) as well as during emotional stimulation with simultaneously passive movement of the affected hand (Hassa et al., 2017) and during cognitive reappraisal (Hassa et al., 2021) was reported. In an earlier fMRI study patients showed hyperactivation of the left insula while trying to move their affected limb compared to HC who simulated the weakness (Stone et al., 2007). These findings go align with the reporting of higher right amygdala and left anterior insula activations during internally and externally generated movements (Voon et al., 2011). In the same study lower activation of the right supplementary motor cortex (SMA) was revealed (Voon et al., 2011). In contrast a higher activation of the SMA during negative emotions in FND patients were reported indicating that there might be a link between emotions and motor dysfunctions (Aybek et al., 2015). This limbic-motor interaction was also shown in the form of increased connectivity in a resting-state analysis between cingulo-insular networks and motor control areas which showed a correlation with symptom severity (Diez et al., 2019, Li et al., 2015a, Li et al., 2015b). Likewise, in our previous study on the same cohort, we detected altered resting-state insular co-activation patterns with the somatomotor- and default mode networks (DMN), which was associated with duration of illness (Weber et al., 2024).

Despite these tremendous advances in the understanding of the role of these brain regions and network connectivity in FND, there is a lack of knowledge on the links between the identified alterations in brain activity and the clinical outcome − the progression or amelioration of the disorder. In a recent positron emission tomography (PET) study, the resting state metabolism of left and right subgenual anterior cingular cortex at inclusion was negatively correlated with improvement of motor symptoms after three months, suggesting this could represent a metabolic state marker (Conejero et al., 2022). Similarly, patients with functional tremor presented not only with a significant improvement in tremor severity but also with a decreased activation in the anterior/paracingulate cortex during a basic-emotion task after 12 weeks of cognitive behavioural therapy (Espay et al., 2019). Furthermore, a study in functional movement disorders patients undergoing a one-week multidisciplinary motor retraining treatment program identified greater primary motor cortex activation at baseline when the patients responded to the therapy program, together with a shift in amygdalar functional connectivity from motor regions towards prefrontal regions (Faul et al., 2020). These studies not only underscore that functional alterations in the brain might revert but can also be detected in response to a clinical improvement. Understanding how brain alterations evolve over time and parallels clinical course could bring valuable information into mechanisms but may also ultimately serve clinical purpose in identifying prognostic biomarkers. Within-group studies are important, as they can account for the coincidence of other neuropsychiatric diseases (Perez et al., 2021) and longitudinal studies are needed to help disentangle between state and trait biomarkers (Conejero et al., 2022; Perez et al., 2021).

Until now, functional alterations in FND were mostly studied using the averaged time-course of blood oxygen level-dependent (BOLD) signals. However, even the resting brain is highly variable in its activity and constantly adapting to its internal and external environment (Brembs, 2021). While in the past particularly this variability of the BOLD signals was considered as noise, it has attracted the attention of researchers in recent years as a more dynamic measurement of brain activity (Garrett et al., 2011). This variability in the BOLD signal is suggested to represent an important feature of proper brain function (Baracchini et al., 2021, Garrett et al., 2018, Garrett et al., 2013) where an optimal variability encompasses a balance that provides enough stability but also the necessary flexibility for the execution of brain functionality (Armbruster-Genç et al., 2016). Therefore, this more dynamic measurement is an option to evaluate the longitudinal changes in brain functioning, which has never been explored in FND so far. For example, motor recovery after stroke showed a correlation with altered temporal variability of the ipsilateral precentral gyrus (Hu et al., 2018). In other neuropsychiatric conditions, there is recent proof of such alterations in brain variability across diverse regions (Kebets et al., 2021, Li et al., 2019, Wei et al., 2023, Zanella et al., 2022): For example, a reduction in BOLD signal variability was associated with improved emotion regulation in with attention/deficit/hyperactivity disorder (ADHD), borderline personality disorder, or bipolar disorder (Kebets et al., 2021, Zanella et al., 2022). Also, patients ADHD presented with overall increased resting-state BOLD signal variability in the prefrontal cortex (Nomi et al., 2018), as well as the sensorimotor- and salience networks (Kebets et al., 2021). Likewise, higher BOLD variability in schizophrenia across diverse brain networks were positively correlated to severity of symptoms (Wei et al., 2023). Moreover.

In this exploratory study, by first examining alterations in BOLD signal variability between FND patients and healthy controls (HC) we aimed to uncover potential signatures of disrupted neural dynamics that may be associated with FND (cross-sectionally) hypothesizing to identify increased BOLD signal variability in diverse brain networks previously associated to FND pathology. Second, we investigated alterations in BOLD signal variability in FND patients over time (longitudinally) to examine alterations in BOLD signal variability with regards to clinical course, hypothesizing that an improvement in symptom severity might align with a reduction in BOLD signal variability in distinct brain regions. Overall, this study aims at identifying both state and prognostic factors of FND.

## 2 Methods

### 2.1 Participants

86 Patients with functional neurological disorder (FND) and 76 healthy controls (HC) participated in a resting-state fMRI study between june 2020 and february 2022, where clinical data were also acquired. Cross-sectional structural and functional imaging data of this cohort (FND patients and HC) have previously been published (Weber et al., 2024, Weber et al., 2022a). FND patients were recruited through the outpatient clinic of the department of Neurology, Inselspital, Bern University Hospital, switzerland. The FND diagnosis was established by a certified neurologist according to DSM-5 criteria (Association and Force, 2013) and ICD-10 (World Health Organization, 2004) of FND (motor FND (F44.4), non-epileptic attack FND (F44.5), sensory FND (44.6) or mixed FND (44.7)) and using positive signs (Stone et al., 2011). Mixed FND was only diagnosed if the patients presented with all three symptom types (motor, sensory and non-epileptic attacks). HC comparable in age and sex were recruited through public advertisement. Inclusion criteria for patients included a diagnosis of FND and an age of minimum 16 years. Exclusion criteria for both groups included: Persons suffering from epilepsy, psychosis, severe major depressive disorder, or alcohol/drug abuse (based on clinical documentation); previously brain surgery; implanted medical devices (pacemaker, infusion pumps); metallic foreign bodies in the head region except braces or dental fillings; breast-feeding or pregnant women; women with the intention to get pregnant; impaired understanding of the task due to language or cognitive difficulties. Board-certified neurologists performed the screening for psychosis, major depressive disorder, and/or drug abuse. Regardless of their clinical status, the patients were invited for a follow-up examination after eight months, at which 53 patients followed this invitation. The reasons for the dropouts are shown in the flowchart in the Supplementary figure S1. The study was approved by the local ethics committee of the canton Bern (SNCTP000002289) and conducted according to the declaration of helsinki.

### 2.2 Clinical assessment

At inclusion (T1), all participants completed the Beck’s Depression Inventory (BDI (Beck et al., 1961)) and the State-Trait Anxiety Inventory (STAI-S and STAI-T (Spielberger et al., 1983)) questionnaires. The State form (STAI-S) assesses the intensity of anxiety experienced by the participant during the test, in the recent past, or anticipates their feelings in a hypothetical scenario whereas the Trait form (STAI-T) looks at individual variations in anxiety predisposition and overall anxiety levels, providing insight into a person's enduring anxious tendencies (Spielberger et al., 1983).

FND patients additionally underwent a clinical examination: Severity of illness was assessed by the Clinical Global Impressions Scale (CGI-I) from 1 to 7 (1 = normal, 7 = among the most ill patients (Busner and Targum, 2007)). In addition, for motor FND symptoms we used the Simplified Functional Movement Disorders Rating scale (S-FMDRS (Nielsen et al., 2017)). At follow-up (T2), we repeated CGI-I and S-FMDRS and additionally assessed the Clinical Global Improvement Score (CGI-II) from 1 to 7 (1 = very much improved, 7 = very much worse). Moreover, we assessed the type of therapy patients engaged in during the four weeks before joining the follow-up measurement.

### 2.3 Image acquisition

MRI data were acquired using a 3 T scanner (Magnetom Prisma, Siemens, Germany). To reduce head movement, the head was fixed using foam cushions. Functional data were acquired using a gradient-echo planar imaging (EPI) sequence with the following parameters: TR = 1.3 s, TE = 37 ms, flip angle = 52°, slice thickness = 2.2 mm, REF voxel size = 2.2x2.2x2.2 mm, TA = 6.39 min and 300 volumes. During the acquisition of the resting-state fMRI, the participants were instructed to lay as calm as possible, to stay awake, to fixate on a cross shown on the screen, and to not think of anything in particular. Structural data were acquired using a T1-weighted MPRAGE sequence with the following parameters: TR = 2.33 s, TE = 3.03 ms, flip angle = 8°, FoV read = 256 mm, 1 mm slice thickness and REF voxel size = 1.0x1.0x1.0 mm, TA = 5.27 min.

### 2.4 Resting-state preprocessing

Preprocessing was performed using Statistical Parametric Mapping version 12 (SPM12; https://www.fil.ion.ucl.ac.uk/spm/software/spm12/). Functional data were corrected for b0-field distortions. The images were realigned and then co-registered to the anatomical image. After this, data was linearly detrended and further denoised using white matter and cerebrospinal fluid signals as well as movement parameters as regressors (including constant, linear, and quadratic trends, average white matter/cerebrospinal fluid time courses, translational and rotational motion time courses upon realignment). Images were normalized to a MNI (Montreal Neurological Institute) template, resampled to 3.0x3.0x3.0 mm and smoothed with a 6 mm FWHM Gaussian kernel. Signals were filtered using a bandpass filter between 0.01 and 0.1 Hz. Functional images were inspected for too high motion artefacts based on Power’s framewise displacement (FD) criterion at a threshold of FD > 0.5 mm (Power et al., 2014).

### 2.5 Differences in BOLD signal variability (SD_BOLD_) between FND and HC

As SD_BOLD_ might represent an adjunctive measure of brain dysfunction in diverse neuropsychiatric disorders (Kebets et al., 2021, Li et al., 2019, Wei et al., 2023, Zanella et al., 2022), we investigated alterations in SD_BOLD_ in FND patients compared to HC on a whole-brain level. Timeseries were mean centred aiming to eliminate the effect of the general activation of the voxels/regions in resting-state (Baracchini et al., 2021). SD_BOLD_ was determined as the voxel-wise standard deviation of the temporal BOLD signal (SD_BOLD_) of each subject, resulting in a 3-dimensional SD_BOLD_ map per subject, as also previously described in (Kebets et al., 2021).

To evaluate differences in the BOLD signal variability between FND and HC a voxel-wise *t*-test was performed using age and gender as variables of no interest. To correct for multiple comparisons, a family-wise error correction (FWE, P < 0.01) was applied at the cluster level. The analysis was repeated using the BDI and STAI-S as additional covariates of no-interest to correct for the effect of depression and anxiety, details are shown in Supplementary Material.

### 2.6 Longitudinal analysis

In a secondary analysis, we investigated on a potential relationship between clinical outcome and SD_BOLD_ in those clusters that were found significantly different between patients and HC. Therefore, we first quantified the results on a network-level by overlaying the significant clusters with the YEO network atlas (Thomas Yeo et al., 2011). For the two most overlapping networks, a region of interest (ROI) was selected based on the AAL2-atlas (Rolls et al., 2015). The average SD_BOLD_ of the voxels in the selected AAL2-regions were extracted from the scans performed at T1 and T2, but only including those voxels from the AAL mask that overlapped with the initial clusters, creating a more granular ROI for subsequent analyses.

As a metric for the evolution of the symptoms between T1 and T2, we calculated the delta-score (Δ) in S-FMDRS and CGI-I by subtracting the score at T1 from the score at T2.

First, we investigated the relationship between symptom severity and SD_BOLD_. Using the imaging data and clinical scores from the initial assessment and the follow-up we calculated the correlation between ΔSD_BOLD_ of the Δ of symptom severity scores (ΔCGI-I, ΔS-FMDRS) using Kendall correlation coefficient, as CGI-I and to some extent S-FMDRS, are ordinal data.

Second, we built a general linear regression model (GLM) to predict the evolution of the clinical scores at T2 (dependent variable) using the SD_BOLD_ values of the individual ROI’s at T1 as independent variable.

## 3 Results

### 3.1 Clinical and demographical data

We excluded one HC and five patients due to excessive movement during the fMRI (N = 5), one patient due to an anatomical brain lesion (N = 1) and one patient due to current drug abuse (N = 1), and one HC did not complete the imaging acquisition, resulting in a sample size of 79 FND patients and 74 HC. There were no significant differences regarding the demographic data of FND patients and HC (see Table 1). FND patients reported significantly higher scores in the BDI and STAI-S (all p-values < 0.001, Table 1).

**Table 1:** Demographic and clinical characteristics between FND patients and healthy controls.

**Characteristic**	**FND**, N = 79	**HC**, N = 74	**p-value**
Age	36.94 (14.31)	33.03 (11.00)	0.17
Gender			0.96
Female	59 (75 %)	55 (74 %)	
Male	20 (25 %)	19 (26 %)	
Depression: BDI; mean (SD)	14.56 (10.25)	3.92 (4.41)	<0.001
Anxiety: STAI 1; mean (SD)	37.42 (11.04)	31.36 (6.32)	<0.001
SF36: General Health; mean (SD)	48.35 (21.36)	79.93 (13.81)	<0.001
S-FMDRS; mean (SD)	7.82 (8.48)	NA	NA
CGI-1; mean (SD)	2.62 (1.59)	NA	NA
Duration; in months; mean (SD)	55.84 (69.30)	NA	NA

Abbreviations: BDI = Beck’s Depression Inventory; STAI = State-Trait Anxiety Inventory, SF-36 = 36-Item Short Form Survey, CGI = Clinical Global impression Score.

As not all patients came back at follow-up, we checked for clinical differences that could represent selection bias. FND patients undergoing follow-up showed no difference in demographic and clinical data compared to FND patients who only participated at inclusion (all p-values < 0.05, Supplementary Table S1). Four patients were excluded for the fMRI analyses due to too high motion artefacts resulting in 49 patients included for the fMRI analysis. From those patients who showed up at the follow-up measurement, most of the patients engaged in one or more kind of therapy (Table 2).

**Table 2:** Therapy type patients engaged in during four weeks before the follow-up.

**Therapy Type**^1^		
Physiotherapy		21 (45 %)
Psychotherapy		27 (57 %)
Occupational therapy		7 (15 %)
Other therapy		15 (32 %)
No therapy		11 (23 %)

**Therapies total**		
	0	11 (23 %)
	1	10 (21 %)
	2	21 (45 %)
	3	4 (8.5 %)
	4	1 (2.1 %)

1 Patients could have engaged in more than one type of therapy. Data from 47 patients on type of therapy was available.

### 3.2 BOLD variability between patients and controls

**Fig. 1:**
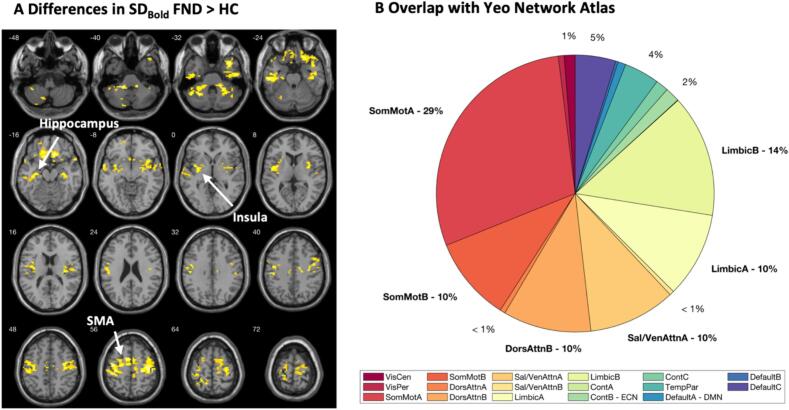
(A) Differences in SD Bold FND > HC showing increased BOLD variability in the hippocampus, the insula and the supplementary motor area (SMA) (corrected for age and gender) and (B) Pie charts illustrating the voxel-wise overlap within the 17 resting-state networks according to (Thomas Yeo et al., 2011). Abbreviations: Cont = Executive control, Default = Default mode DorsAttn = Dorsal attention, Sal/VenAttn = Salience/Ventral attention, SomMot = somatomotor, TempPar = Temporoparietal, VisCen = Central vision, VisPer = Peripheral Visual, SMA = Supplementary Motor Area.IN COLOUR, 2-COLUMN FITTING.

Following the exclusion of subjects with too high motion artefacts, the FND group still differed in terms of number of discarded volumes from HC (FND 5.68 % versus HC 1.61 %, Z =  − 5.1, *P* < 0.001) while no differences were found terms of total FD. Corrected for age and sex, the differences in SD_BOLD_ between FND and HC revealed six significant clusters where FND patients showed higher SD_BOLD_ compared to HC across brain regions including the insula, the hippocampus, the supplementary motor area (SMA), the orbitofrontal cortex and the cerebellum (Fig. 1). The results survived correction for family-wise error on cluster level. Characteristics of these clusters are shown in Supplementary Table S2. The mapping to the YEO network atlas showed, that the voxels within these clusters were mostly overlapping with the somatomotor network (39 %), the attention/salience networks (22 %), and the limbic network (24 %). These results remained largely stable when correcting for anxiety and depression (Supplementary Material, Fig. S2, Table S4).

### 3.3 Evolution of symptom severity associated with BOLD variability

Patients did not differ between M0 and M8 in terms of number of discarded volumes or total FD. To investigate the relationship between BOLD variability and clinical outcomes in FND patients, we extracted the SD_BOLD_ from the main hubs of the three most predominant networks found to be altered in patients compared to HC. For the somatomotor network we selected the SMA, and for the attention networks the insula.

**Fig. 2:**
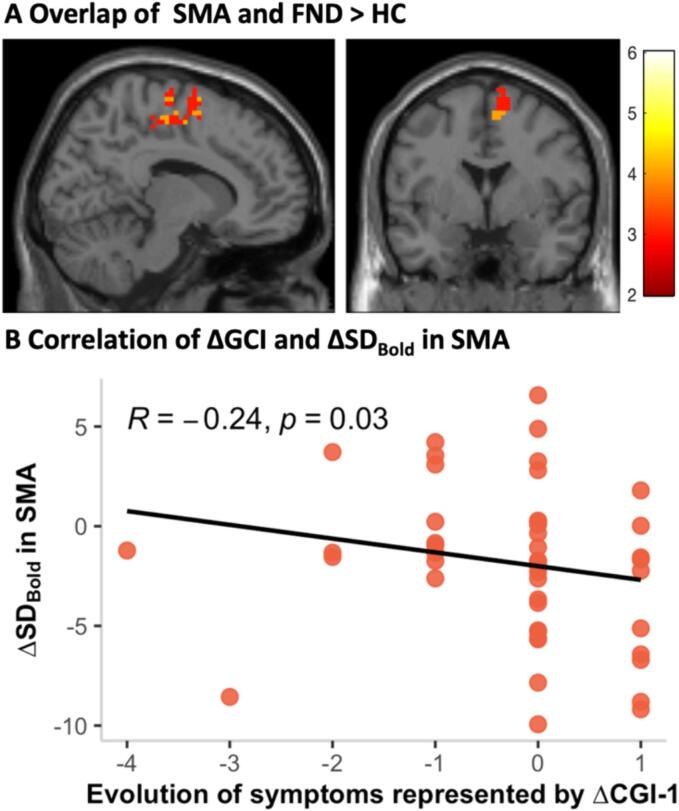
Correlation of ΔCGI and ΔSD_BOLD_ in SMA. (**A**) Overlap of SMA with the contrast of FND > HC (corrected for age and gender). (**B**) Correlation plot of ΔCGI and ΔSD_BOLD_ in SMA. The ΔCGI-1 ranges from −6 to + 6 with a positive ΔCGI-1 representing a worsening in symptom severity and a negative ΔCGI-1 representing an improvement of the symptom severity. IN COLOUR, 1.5-COLUMN FITTING.

The correlation of the Δsymptom severity and ΔSD_BOLD_ showed a significant negative correlation between ΔCGI-1 and the SMA. With the CGI-1 ranging from one (no symptoms) to seven (among most extremely ill patients), the ΔCGI-1 can range from −6 to + 6 with a positive ΔCGI-1 meaning a worse general impression was reported at T2 compared to T1, and a negative ΔCGI-1 meaning an improved general impression was reported at T2 compared to T1. A positive ΔSD_BOLD_ means a higher SD_BOLD_ at T2 compared to T1, and vice versa. Together this indicates that an improvement of the symptom severity represented by a negative ΔCGI-1 correlates with an increased SD_BOLD_ in the SMA at T2 compared to T1 represented by a positive ΔSD_BOLD_ (Fig. 2). There were no significant correlations with ΔS-FMDRS.

In the predictive GLM the SD_BOLD_ in the left Insula could predict ΔCGI-1 (*ß* = 0.1, *P* = 0.041). Thus, a higher SD_BOLD_ at T1 was linked to a positive ΔCGI-1 at T2 indicating a worsening of the symptoms. The were no significant predictions for the S-FMDRS. Results were controlled for age and gender.

## 4 Discussion

In a cross-sectional as well as longitudinal design, our study aimed to investigate the spatial patterns of BOLD signal variability in FND that may be associated with the severity of symptoms and their potential role as a biomarker for this complex condition. Compared to HC, FND patients presented higher BOLD signal variability in key brain networks, including the somatomotor, salience, and limbic networks, covering regions such as the insula, the hippocampus, and the SMA as well as the orbitofrontal cortex. These findings align with previous neuroimaging research on FND emphasizing aberrant neural activity and disrupted functional connectivity in static measurements (Drane et al., 2021; Perez et al., 2021; Pick et al., 2019) adding a novel dynamic dimension to our understanding of underlying mechanisms.

### 4.1 Critical interplay between somatomotor, salience and limbic networks in FND

In FND, altered network connectivity between the somatomotor, salience and limbic networks have frequently been identified and brought in context with the symptomatology of FND suggesting interrelated mechanisms. Functional alterations within these networks were suggested to interfere with a proper preparation and execution of motor functions (Cojan et al., 2009) often associated with emotional arousal (Aybek et al., 2015), combined with an impaired integration of sensory information (Huys et al., 2021, Pareés et al., 2012), thus depicting FND as a large-scale brain network dysfunction (Perez et al., 2012).

The SMA is a key region of the somatomotor network and is involved in motor planning and execution and has been implicated in FND symptom generation (Aybek et al., 2015; Stone et al., 2007). Moreover, the insula as an important hub of the salience network has been implicated in various cognitive and affective processes, including interoception (Haruki and Ogawa, 2021), emotion regulation (Gasquoine, 2014), and self-awareness (Modinos et al., 2009, Tisserand et al., 2023), for which alterations within these cognitive processes have been previously reported in FND (Sojka et al., 2021, Sojka et al., 2018). Moreover, our previous results derived from the same cohort showed altered insular co-activation patterns with the somatomotor as well as default mode network in FND, which was associated with duration of illness as well as stress biomarkers (Weber et al., 2024). Abnormal emotion regulation in FND was previously directly linked to motor outputs: As such, decreased activity was found in the insula and motor regions in patients with functional dystonia in an emotional face fMRI task (Espay et al., 2018b). Further task-based fMRI studies showed that when asked to maintain a grip force while pleasant and unpleasant images were shown, patients showed an amplified force compared to HC towards unpleasant images, together with increased activity in the hippocampus and the posterior cingulate cortex (PCC) (Blakemore et al., 2016). The PCC and the hippocampus are thought to be involved in self-reflective behaviour (Brewer et al., 2013), and their aberrant activity was attributed to enhanced evaluation of visual stimuli as emotionally relevant. Similarly, when recalling traumatic life events during fMRI, FND patients showed amongst others increased activity of the SMA and the dorsolateral prefrontal cortex (dlPFC), together with decreased activity in the hippocampus (Aybek et al., 2014). Moreover, increased functional connectivity between the SMA and the amygdala was identified (Aybek et al., 2014). Particularly, the dlPFC together with the SMA are involved in motor planning and selection of actions based on internal and external cues and emotional states (Dixon et al., 2017, Hoffmann, 2013, Nachev et al., 2008), while the hippocampus as a key node of the DMN plays an important role in emotion-associated memory processing (Yang and Wang, 2017). In summary, it was firstly shown that limbic influence in patients might modulate voluntary motor actions, suggesting a tight interplay between limbic, salience and somatomotor network in FND symptomatology.

To our knowledge this is the first study investigating on BOLD signal variability in patients with FND, which further supports and extends previous findings. In general, brain signal variability offers valuable insights into brain activity unrelated to traditional measures of BOLD activation measurements (Depue et al., 2010, Garrett et al., 2010). While optimal brain function necessitates a certain level of variability, excessively high variability may be detrimental to the efficiency of inhibition of distractions and cognitive stability (Armbruster-Genç et al., 2016). In particular, the existence of a connection between symptom severity and brain variability has been demonstrated in other neuropsychiatric disorders. In ADHD increased symptom severity was related to increased resting-state brain variability in the dorsal and ventral medial prefrontal cortex (Nomi et al., 2018). Also in depression and mania a correlation of the clinical scores of symptoms and brain variability in the DMN and somatomotor network was revealed (Martino et al., 2016). In schizophrenia higher BOLD variability in the language-, dorsal attention- and auditory networks and lower BOLD variability in the DMN, executive control-, somatosensory- and visual networks showed a positive correlation with the severity of positive and negative symptoms (Wei et al., 2023). As such, the increased BOLD signal variability as found particularly in the somatomotor and salience networks in FND patients might affect cognitive stability leading to over-reactivity of neural circuits (Kebets et al., 2021) which might further destabilize a proper planning of motor actions leading to functional neurological symptoms.

In summary, this study not only supports previous findings but also adds another dimension to the study of brain functional alterations in patients with FND. As such, it highlights the critical interplay between somatomotor- and salience networks in patients with FND, suggesting that higher BOLD signal variability in these regions/networks might contribute to the pathophysiology of FND.

### 4.2 BOLD signal variability in the supplementary motor area aligns with clinical outcome

Further evidence arises from our longitudinal data. The results of our correlation analysis revealed an increase in BOLD signal variability in the SMA over time in subjects who had an improved clinical outcome after eight months. In other words, a lower variability might contribute to the manifestation of FND symptoms, while an increase in variability in the SMA aligns with the clinical improvement in these patients. This finding is contrary to previous studies in neuropsychiatric disorders investigating BOLD signal variability in which a clinical improvement was most commonly in line with a reduction of BOLD signal variability (Kebets et al., 2021, Martino et al., 2016, Nomi et al., 2018, Wei et al., 2023). However, our findings of an improvement in symptom severity in line with an increase in BOLD signal variability in the SMA corroborates the previous notion that both excessive as well as insufficient BOLD signal variability could detrimentally impact proper brain functioning (Armbruster-Genç et al., 2016, Baracchini et al., 2021, Garrett et al., 2018), thereby underscoring the significance of maintaining a balanced variability.

Moreover, we found that higher BOLD signal variability at baseline (T1) in the left insula was associated with the evolution of clinical symptoms, indicating that patients who in general show higher BOLD signal variability in the insula were less likely to show an improved outcome after eight months. The insula with its subregions serves as a hub for integrating sensory, emotional, and cognitive information, contributing to the regulation and understanding of emotions (Centanni et al., 2021). Higher brain signal variability in the sensorimotor and salience networks − including the insula − and lower variability in the DMN (Kebets et al., 2021) were associated with a better emotion regulation in ADHD disorder, bipolar disorder and borderline personality disorder. Another study found that higher BOLD variability in the sensorimotor and salience network could predict the use of reappraisal strategy compared to the prediction of using emotion suppression by a decreased variability in the salience network (Zanella et al., 2022). In the context of FND, where impaired emotion regulation is recognized as a key pathophysiological mechanism (Krámská et al., 2020), the heightened variability of the insula may signify a dysregulation of these processes. This dysregulation could potentially contribute to the persistence or worsening of symptoms over time.

Up to date, only a few studies investigated whether brain functional alterations in FND patients are directly linked to the dynamic of functional symptoms (state marker), how it parallels fluctuations and/or maintenance of symptoms and if these alternations can represent prognostic factors. Using stepwise FC analyses on resting-state data of FND patients, Diez and colleagues identified enhanced functional propagation from primary motor areas to the amygdala, the insula, the cingulate cortex, as well as the temporo-parietal junction. Moreover, functional propagation profiles of the insula and the amygdala correlated with symptom severity and could predict clinical improvement after a six-months follow-up (Diez et al., 2019). Closely aligning with our results, a previous study using PET-imaging identified a hypometabolism in the SMA in FND patients which disappeared after a three-month follow-up in patients with an improved clinical outcome (Conejero et al., 2022). Likewise, the metabolism in the anterior cingulate cortex (ACC) at inclusion strongly correlated with clinical improvement after three months (Conejero et al., 2022). The authors thus suggested the existence of a metabolic state and prognostic marker for FND associated with motor symptoms and recovery.

These findings together with ours, represent a novel way to consider neuroimaging as potentially useful in clinical settings for FND. This search for biomarkers is needed not for diagnostic purposes as clinical signs have been found reliable and diagnostic criteria are established but because it is still difficult to predict which patient will have a favourable outcome (Gelauff et al., 2019). Having a prognostic biomarker at disease onset helping predict outcome may become useful when delivering targeted treatment and triaging patients into care pathways (Finkelstein et al., 2023), having a state marker of disease improvement may become useful in research setting for clinical trials. Our results suggest that dynamic changes in BOLD variability in the SMA may be a state marker of disease progression while the increased variability in the insula may serve as a prognostic marker for clinical outcome. Both warrant further replication in independent samples before they could be used in the advancing field of clinical research developing targeted intervention (Conejero et al., 2022; Perez et al., 2021).

## 5 Limitations

It is important to note that our study has some limitations. First, as this is the first study looking at BOLD variability in FND patients it is important to consider other reasons for a higher BOLD variability. FND patients show a high comorbidity with depression and anxiety disorder (Butler et al., 2021) which may influence the results. However, when including depression and anxiety as covariates of no-interest to our model (see Supplementary Material), our results remained largely stable. In generalized anxiety disorder patients presented with a decreased brain signal variability in widespread networks and regions such as the visual-, sensorimotor-, and frontoparietal networks, the limbic system, and the thalamus (Li et al., 2019). As these findings are contrary to ours it is unlikely that anxiety is a major confounder in our study. However, the different direction of these results might represent another underlying mechanism. Moreover, it needs to be acknowledged that anxiety is a common comorbidity in FND which might add another level of complexity to the underlying pathophysiological mechanisms(Ludwig et al., 2018). Despite the board-certified neurologists screened the patients for concomitant psychiatric disorders, no systematic psychiatric evaluation was performed. Similarly, psychotropic medication was only assessed at T1, thus the longitudinal results could not be corrected for psychotropic medication intake. Second, while the here reported results provide important information of potential state marker of disease improvement, it must be acknowledged that the reported correlation between change in symptom severity and change in SD_Bold_ in the SMA is weak (*R* = -0.24), albeit significant. Moreover, as this represents a novel approach in FND, these findings necessarily must be replicated and validated in other cohorts and potential subgroups. Third, cardiovascular factors such as heart rate may also influence the variability of BOLD signal, together with neural factors they can explain the changes of BOLD signal variability in ageing (Tsvetanov et al., 2021). As we had a control group comparable in age and sex, these effects should be negligible. As this method has not been explored in FND these findings need to be interpreted with caution and further studies using the same approach are necessary to confirm our results. Fifth, there exits several methods (Waschke et al., 2021) analysing brain signal variability, and using a different approach could lead to different results. Likewise, a ROI approach was applied for the longitudinal analyses. While including only those voxels that overlapped in the ROI and the cluster, a more fine-grained ROI or creating seeds based on peak differences in the group-level analyses might provide different results. Lastly, the between-group analysis (FND versus HC) baseline findings were used to narrow the search window for the longitudinal analyses. Despite of the whole-brain analyses did not bear significant results (Supplementary Material), such an approach might cause that important findings may not be well accounted for. While our cohort represents patients with mixed FND symptoms, stratifying patients into different symptom types or different outcome groups could have informed the results differently.

## 6 Conclusion

In conclusion, our study provides evidence of altered BOLD signal variability in specific brain networks in FND encompassing the somatomotor, limbic and salience networks. These findings add to previous literature supporting the notion that FND might be depicted as a large-scale brain network dysfunction in which salience and limbic networks tightly interplay with somatomotor networks potentially affecting motor planning and execution. Moreover, the SMA variability may serve as a state marker, given that a change in BOLD signal variability corresponded to a clinical improvement. Furthermore, the insula demonstrates its potential as a prognostic marker, at which patients with higher BOLD signal variability at inclusion (T1) were less likely to show a clinical improvement. These finding contribute to the growing understanding of FND pathophysiology and highlight the potential for BOLD signal variability as a promising method for further research.

## 7 Funding/support

This work was supported by the Swiss National Science Foundation (SNF Grant PP00P3_176985 for SA) and the University Hospital Inselspital Bern, Switzerland.

## CRediT authorship contribution statement

**Ayla Schneider:** Writing – review & editing, Writing – original draft, Visualization, Methodology, Formal analysis, Conceptualization. **Samantha Weber:** Writing – review & editing, Writing – original draft, Validation, Supervision, Software, Project administration, Methodology, Investigation, Formal analysis, Data curation, Conceptualization. **Anna Wyss:** Data curation, Validation. **Serafeim Loukas:** Software, Methodology, Formal analysis. **Selma Aybek:** Writing – review & editing, Supervision, Resources, Funding acquisition.

## Declaration of Competing Interest

The authors declare that they have no known competing financial interests or personal relationships that could have appeared to influence the work reported in this paper.

## Data Availability

Data will be made available on request.
