# Supplementary material for: BOLD signal variability as potential new biomarker of functional neurological disorders
Source: Neuroimage Clin. 2024 May 31;43:103625. doi: 10.1016/j.nicl.2024.103625 (PMC11179625; doi:10.1016/j.nicl.2024.103625)
Supplement: Supplementary Data 1 [file mmc1.pdf]

Supplementary Material

BOLD signal variability as potential new biomarker of functional neurological disorders

Authors: Ayla Schneider[a,b†], Samantha Weber[a,b,c†], Anna Wyss[a,d,],Serafeim Loukas[a,e], Selma Aybek[a,f]

Author Affiliations:
[a]Department of Neurology, Psychosomatic Medicine Unit, Inselspital Bern University Hospital, University of Bern, 3012 Bern, Switzerland
[b]Translational Imaging Center (TIC), Swiss Institute for Translational and Entrepreneurial Medicine, 3010 Bern,Switzerland.
[c]University of Zurich, Psychiatric University Hospital Zurich, Department of Psychiatry, Psychotherapy and Psychosomatics, 8032 Zurich, Switzerland.
[d]Graduate School for Health Sciences (GHS), University of Bern, 3006 Bern, Switzerland.
[e]Institute of Bioengineering, Ecole Polytechnique Fédérale de Lausanne (EPFL), 1015 Lausanne, Switzerland
[f]Faculty of Science and Medicine, University of Fribourg, 1700 Fribourg, Switzerland

†These authors contributed equally.

Supplementary Methods

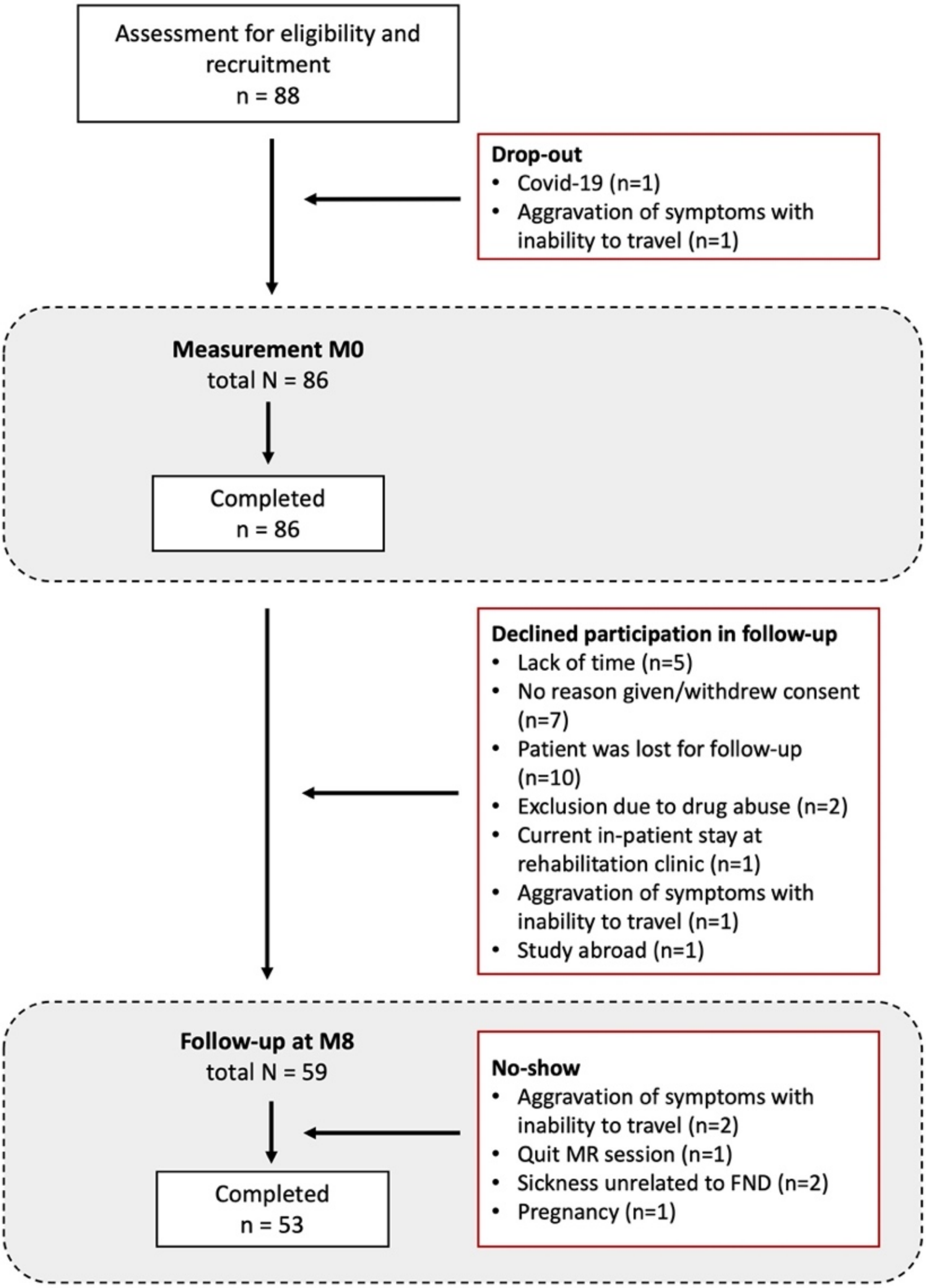

Fig. S1. Flowchart illustrating the dropouts and number of subjects excluded based on different criteria during initial assessment and follow up after 8 months.

Control analyses

A control analysis was performed to assess the robustness of our results, whereby we performed the same analysis for the differences in BOLD signal variability between FND and HC with the correction of the effect of depression, anxiety, and psychotropic medication (dichotomized into yes/no including one or more of the following: benzodiazepines, antidepressants neuroleptics, antiepileptics, opioids). None of the HC used psychotropic medication at the time of the study. 32 patients were under current psychotropic medication. To do so, we used BDI, STAI-S and psychotropic medication as additional covariates of no-interest in the voxel-wise t-test. We also quantified the results on a network-level by overlaying significant clusters with the YEO network atlas.

Whole brain longitudinal analysis

To firstly look of the evolution of SD_{BOLD} from T1 to T2 in FND patients, a voxel-wise t-test was performed using age and gender as variables of no interest comparing the scans from T1 to scans at T2. To correct for multiple comparisons, a family-wise error correction (FEW, $P < 0.01$) was applied at the cluster level

Supplementary Results

Table S1 Demographic and clinical features at baseline of individuals with functional neurological disorder (FND) who engaged in follow-up compared to those who did not participate. Abbreviations: FUP = Patients who participated in the follow-up, Non-FUP = Patients who did not participate in the follow-up.

**Characteristic**	**FUP**, N = 49	**Non-FUP**, N = 30	**p-value**
Age	36.92 (14.34)	36.97 (14.52)	0.911
Gender			0.751
Female	36 (73%)	23 (77%)	
Male	13 (27%)	7 (23%)	
Depression: BDI	13.39 (10.00)	16.47 (10.53)	0.179
Anxiety: STAI-S	35.86 (10.66)	39.97 (11.36)	0.109
SF36: General Health	49.90 (20.53)	45.83 (22.78)	0.547
S-FMDRS	7.29 (7.86)	8.70 (9.48)	0.603
Clinical General Impression 1	2.51 (1.61)	2.80 (1.56)	0.388
Symptom Duration	55.34 (59.76)	56.66 (83.70)	0.315

Table S2: Significant Clusters of voxel-wise contrast analysis FND > HC

Cluster-level			Peak-level						
P_{FWE}	P_{FDR}	Cluster extent	P_{FWE}	P_{FDR}	Peak voxel Z-score	Peak coordinates in MNI Space x,y,z {mm}			cerebral regions
<0.001	<0.001	1944	0.002	0.045	5.4	-39	-31	59	Left postcentral gyrus
			0.002	0.045	5.37	30	-22	53	Right precentral gyrus
			0.007	0.054	5.17	-39	-10	56	Left precentral gyrus
0.000	0.000	1053	0.004	0.051	5.26	-54	11	-13	Left temporal pole: superior temporal gyrus
			0.01	0.054	5.09	12	26	-16	Right gyrus rectus
			0.019	0.055	4.97	60	14	-13	Right temporal pole: superior temporal gyrus
0.000	0.000	303	0.033	0.066	4.86	-48	-55	-34	Left Cerebellum
			0.061	0.101	4.72	-27	-58	-31	Left Cerebellum
			0.067	0.103	4.7	-27	-58	-55	Left Cerebellum
0.000	0.000	124	0.128	0.113	4.53	24	-46	-31	Right Cerebellum
			0.207	0.126	4.4	18	-37	-31	Right Cerebellum
			0.533	0.242	4.09	45	-61	-34	Right Cerebellum
0.003	0.007	81	0.139	0.114	4.51	21	-67	-55	Right Cerebellum
			0.205	0.126	4.41	21	-58	-55	Right Cerebellum
			1	0.786	3.27	45	-61	-58	Right Cerebellum
0.040	0.066	47	0.193	0.126	4.42	-18	-91	-4	Left calcarine fissure and surrounding cortex
			0.901	0.381	3.77	-42	-85	-10	Left inferior occipital gyrus
			1	0.787	3.26	-15	-94	5	Left calcarine fissure and surrounding cortex & Left superior occipital gyrus
0.032	0.060	50	0.909	0.381	3.76	27	-85	2	Right middle occipital cortex
			0.957	0.438	3.67	15	-76	-7	Right lingual gyrus
			0.967	0.457	3.65	15	-79	8	Right calcarine fissur and surrounding cortex

Table S3: Percentage of voxels in the significant cluster of the selected AAL2 regions.

AAL2	Percentage
SMA right	6.31%
SMA left	11.43%
Insula left	6.54%
Insula right	3.34%
Hippocampus left	13.19%
Hippocampus right	10.42%

Control analyses – Corrected for age, gender, depression, anxiety and psychotropic medication

The analysis of the differences in SD_{BOLD} between FND and HC revealed 3 significant clusters ($p_{FWE} < 0.05$, minimum cluster size = 50 Voxels) and one cluster showing a trend ($p_{FWE} = 0.061$, cluster size = 42 voxels), where FND patients showed higher SD_{BOLD} compared to HC across brain regions including the insula, the supplementary motor cortex (SMA) and basal ganglia (Fig. S2). Characteristics of these clusters are shown in Table S4. 61% percent of the voxels in the control analysis were overlapping with the original analysis. The mapping to the YEO network atlas showed, that the voxels within these clusters were mostly overlapping with the limbic network (55%), somatomotor network (16%) and default mode network (22%).

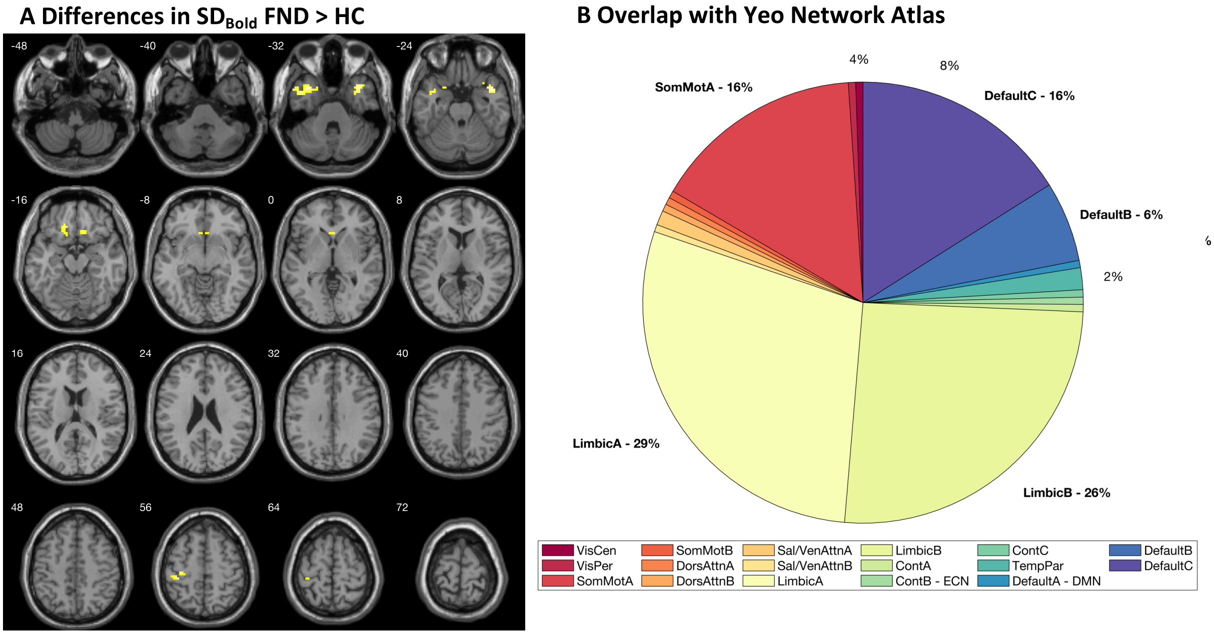

Fig. S2 (**A**) Differences in SD Bold FND > HC showing increased BOLD variability in the insula, the supplementary motor area (SMA) und the basal ganglia (corrected for age, gender, depression [BDI], anxiety [STAI] and and psychotropic medication) (**B**) Pie charts illustrating the voxel-wise overlap within the 17 resting-state networks according to the convention of Yeo. Abbreviations: Cont = Executive control, Default = Default mode DorsAttn = Dorsal attention, Sal/VenAttn = Salience/Ventral attention, SomMot = somatomotor, TempPar = Temporoparietal, VisCen = Central vision, VisPer = Peripheral Visual, SMA = Supplementary Motor Area.

Table S4: Significant clusters of FND > HC in control analysis corrected for age, gender, depression (BDI), anxiety (STAI-1) and psychotropic medication.

Cluster				Peak						
P_{FWE}	P_{FDR}	p_{uncorr}	cluster extent	P_{FWE}	P_{FDR}	Peak voxel Z-score	Peak coordinates in MNI Space x,y,z {mm}			cerebral regions
0.010	0.046	0.001	64	0.082	0.500	4.65	-51	8	-28	Left middle temporal gyrus
				0.173	0.500	4.45	-33	11	-28	Left superior temporal gyrus
				0.383	0.5000	4.21	-39	5	-28	Left superior temporal gyrus
0.029	0.002	<0.001	51	0.115	0.500	4.56	45	8	-25	Right superior temporal gyrus
				0.246	0.500	4.35	39	11	-31	Right superior temporal gyrus
				0.994	0.885	3.51	27	11	-22	Right Insula
0.061	0.004	<0.001	42	0.319	0.500	4.27	-29	-31	59	Left postcentral gyrus
				0.501	0.500	4.12	-33	-22	56	Left precentral gyrus
				0.789	0.722	3.89	-45	-25	-13	Left postcentral gyrus
0.002	<0.001	<0.001	84	0.506	0.500	.11	-9	23	-13	Left ACC
				0.551	0.500	4.08	-15	32	-16	Left medial orbital gyrus
				0.762	.699	3.91	12	23	-16	Right gyrus rectus

Whole brain longitudinal analysis

The analysis of the differences in SD_{BOLD} between start of the study and follow-up revealed 3 significant clusters ($P_{FWE} < 0.05$, minimum cluster size = 50 voxels), where FND patients at follow-up showed higher SD_{BOLD} compared to inclusion.

Table S5: Significant clusters of T2 > T1 in control analysis corrected for age and gender

Cluster				Peak						
P_{FWE}	P_{FDR}	p_{uncorr}	cluster extent	P_{FWE}	P_{FDR}	Peak voxel Z-score	Peak coordinates in MNI Space x,y,z {mm}			cerebral regions
0.010	0.046	0.001	64	0.082	0.500	4.65	-51	8	-28	Left middle temporal gyrus
				0.173	0.500	4.45	-33	11	-28	Left superior temporal gyrus
				0.383	0.5000	4.21	-39	5	-28	Left superior temporal gyrus
0.029	0.002	<0.001	51	0.115	0.500	4.56	45	8	-25	Right superior temporal gyrus
				0.246	0.500	4.35	39	11	-31	Right superior temporal gyrus
				0.994	0.885	3.51	27	11	-22	Right Insula
0.061	0.004	<0.001	42	0.319	0.500	4.27	-29	-31	59	Left postcentral gyrus
				0.501	0.500	4.12	-33	-22	56	Left precentral gyrus
				0.789	0.722	3.89	-45	-25	-13	Left postcentral gyrus
0.002	<0.001	<0.001	84	0.506	0.500	.11	-9	23	-13	Left ACC
				0.551	0.500	4.08	-15	32	-16	Left medial orbital gyrus
				0.762	.699	3.91	12	23	-16	Right gyrus rectus

The correlation of the Δsymptom severity and ΔSD_{BOLD} showed a significant negative correlation between ΔCGI-1 and the SMA. A positive ΔCGI-1 means a worse general impression was reported at T2 compared to T1, a positive ΔSD_{BOLD} means a higher SD_{BOLD} at T2 compared to T1. Together this indicates that an improvement of the symptom severity represented by a negative ΔCGI-1 correlates with an increased SD_{BOLD} in the SMA at T2 compared to T1 represented by a positive ΔSD_{BOLD} (Fig. 2). There were no significant correlations with ΔS-FMDRS.

In the predictive GLM (corrected for anxiety and depression) the SD_{BOLD} in the left insula could predict ΔCGI-1 (ß= 0.11, p-value = 0.025), as well as in the right insula (ß= 0.47, p-value = 0.045). Thus, a higher SD_{BOLD} at T1 was linked to an improvement of the CGI-1. There were no significant predictions for the S-FMDRS.

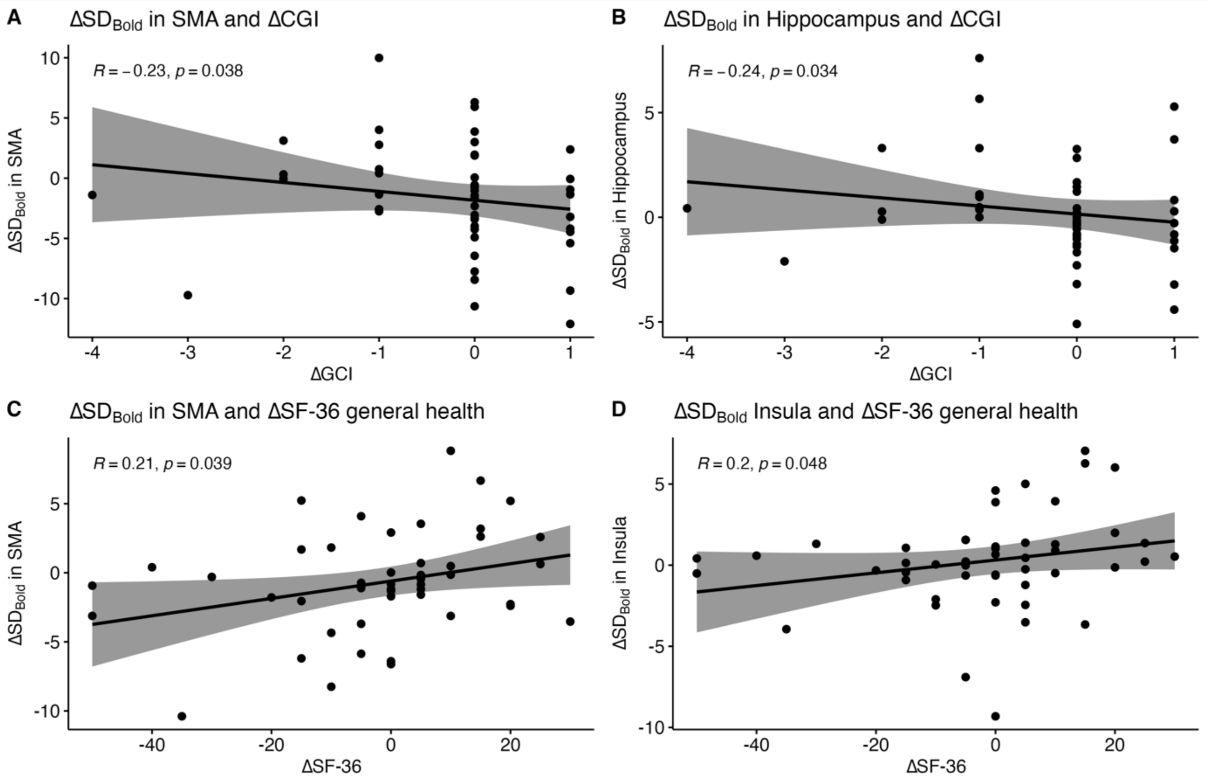

Fig. S4: Correlation of clinical scores and ΔSD_{BOLD} in different Brain regions. (A, B) Correlation of ΔSD_{BOLD} in the right SMA/right Hippocampus and ΔCGI. **(C,D)** Correlation of of ΔSD_{BOLD} in the left SMA/left Insula and ΔSF-36 general health.
